# Excess mortality among people with podoconiosis: secondary analysis of two Ethiopian cohorts

**DOI:** 10.1093/trstmh/traa150

**Published:** 2020-11-24

**Authors:** Hannah Masraf, Temesgen Azemeraw, Meseret Molla, Christopher Iain Jones, Stephen Bremner, Moses Ngari, James A Berkley, Esther Kivaya, Greg Fegan, Abreham Tamiru, Abebe Kelemework, Trudie Lang, Melanie J Newport, Gail Davey

**Affiliations:** Centre for Global Health Research, Brighton and Sussex Medical School, University of Sussex, Falmer, Brighton BN1 9PX, UK; Dabat Research Centre, University of Gondar, Gondar, Ethiopia; School of Public Health, Addis Ababa University, PO Box 9086, Addis Ababa, Ethiopia; Department of Primary Care and Public Health, Brighton and Sussex Medical School, University of Sussex, Brighton BN1 9PX, UK; Department of Primary Care and Public Health, Brighton and Sussex Medical School, University of Sussex, Brighton BN1 9PX, UK; KEMRI/Wellcome Trust Research Programme, Kilifi, Kenya; KEMRI/Wellcome Trust Research Programme, Kilifi, Kenya; Centre for Tropical Medicine and Global Health, University of Oxford, Oxford, UK; KEMRI/Wellcome Trust Research Programme, Kilifi, Kenya; KEMRI/Wellcome Trust Research Programme, Kilifi, Kenya; Swansea University Medical School, Swansea, UK; International Orthodox Christian Charities Podoconiosis Project, Debre Markos, Ethiopia; International Orthodox Christian Charities Podoconiosis Project, Debre Markos, Ethiopia; KEMRI/Wellcome Trust Research Programme, Kilifi, Kenya; Centre for Tropical Medicine and Global Health, University of Oxford, Oxford, UK; Centre for Global Health Research, Brighton and Sussex Medical School, University of Sussex, Falmer, Brighton BN1 9PX, UK; Centre for Global Health Research, Brighton and Sussex Medical School, University of Sussex, Falmer, Brighton BN1 9PX, UK; School of Public Health, Addis Ababa University, PO Box 9086, Addis Ababa, Ethiopia

**Keywords:** Ethiopia, mortality, non-filarial elephantiasis, podoconiosis

## Abstract

**Background:**

While morbidity attributable to podoconiosis is relatively well studied, its pattern of mortality has not been established.

**Methods:**

We compared the age-standardised mortality ratios (SMRs) of two datasets from northern Ethiopia: podoconiosis patients enrolled in a 1-y trial and a Health and Demographic Surveillance System cohort.

**Results:**

The annual crude mortality rate per 1000 population for podoconiosis patients was 28.7 (95% confidence interval [CI] 17.3 to 44.8; n=663) while that of the general population was 2.8 (95% CI 2.3 to 3.4; n=44 095). The overall SMR for the study period was 6.0 (95% CI 3.6 to 9.4).

**Conclusions:**

Podoconiosis patients experience elevated mortality compared with the general population and further research is required to understand the reasons.

## Introduction

Many of the neglected tropical diseases (NTDs) listed for elimination, including soil-transmitted helminthiases, schistosomiasis, lymphatic filariasis (LF), onchocerciasis and trachoma, are thought of as disabling rather than the direct causes of excess mortality. Disability-adjusted life years (DALYs) estimates for these diseases are predominantly influenced by years lived with disability rather than years of life lost, although this may reflect the absence of data on mortality rather than data on the absence of mortality.^1^

Podoconiosis, a non-infectious, geochemical NTD characterised by lymphoedema, is associated with severe social, economic, physical and mental health consequences.^2^ Acute dermatolymphangioadenitis (ADLA) attacks, similar to those in LF patients, occur at least monthly among untreated patients.^3^ Stigma results in the inability to marry, divorce among those already married, exclusion from school and ostracism in the community. Disability causes a productivity loss of 45% of working days per year on average, and depression is common.^2^ No information on mortality has yet been reported, although anecdotal evidence suggests increased mortality related to acute attacks.

We compared mortality in a group of podoconiosis patients enrolled in a clinical trial with that in a local general population cohort. Podoconiosis patient data were obtained from the completed Gojjam Lymphoedema Best Practice Trial (GoLBeT).^4^ In brief, this trial tested the effectiveness of a simple lymphoedema self-care package in reducing the incidence of ADLA among podoconiosis patient participants who were followed up over a 12-month period. This took place in the Aneded *woreda* (district) of Amhara region, 278 km northwest of Ethiopia's capital Addis Ababa and 260 km from Bahir Dar, Amhara's regional capital. GoLBeT mortality rates were standardised against those of the Dabat Health and Demographic Surveillance System (HDSS), a vital event registration system in the Dabat district of Amhara, 821 km northwest of Addis Ababa.^5^

## Methods

The GoLBeT dataset and the Dabat HDSS were retrospectively analysed using data from a 17-month period (28 February 2015–30 July 2016) to ensure comparability. The Dabat HDSS was selected as the reference population, representing the Amhara region's general population, as it shares sociodemographic characteristics with the GoLBeT and, like Aneded, Dabat's economy relies on subsistence farming.

Demographic data including age, sex, and family-reported date of death were used in this analysis. Data from outside of the 17-month study period and people with incomplete demographic data and <18 y of age were excluded, thus aligning this with the GoLBeT inclusion, exclusion and withdrawal criteria.

Data analysis was performed with SPSS version 25.0 (IBM, Armonk, NY, USA). The age distributions of both populations were descriptively analysed and compared using a two-tailed Mann–Whitney U test. The crude and age-standardised mortality rates were calculated and expressed per 1000 population, using the total number of deaths in the population as the numerator and the total population size as the denominator. The Dabat HDSS mortality rates were then annualised. The 95% confidence intervals (CIs) for the annual crude and age-adjusted rates were calculated using the Poisson distribution. SMRs were calculated using indirect standardisation. CIs for the SMRs were obtained using Fisher's exact test, due to the small expected number of deaths.

## Results

A total of 696 people were randomised to immediate or delayed treatment arms in the GoLBeT. However, 33 withdrew before completion or lacked adequate data from which to compute mortality rates and were excluded.^4^ Thus a total of 663 participants were included: 344 males (51.9%) and 319 females (48.1%) with a median age of 50.0 y (interquartile range [IQR] 40.0–60.0).

The Dabat dataset contained 229 484 entries for 88 041 individuals. Once the inclusion/exclusion criteria were applied, 44 095 individuals were suitable for analysis: 20 927 males (47.5%) and 23 168 females (52.5%). The median age was 31.0 y (IQR 23.0–45.0). The age distributions in the datasets differed, with the GoLBeT population being older (p<0.001), but the sex distributions were similar, with a 4.4% difference. For full demographic data see Supplementary Table 1.

There were 19 deaths in the GoLBeT population and 175 deaths in the Dabat HDSS. The crude annual mortality rate for the GoLBeT dataset was 28.66 (95% CI 17.25 to 44.75) per 1000 population, while that of the general population was 2.79 (95% CI 2.27 to 3.39) per 1000 population. The crude mortality rate ratio (MRR) was 10.26 (95% CI 3.00 to 35.05), indicating overall excess mortality among podoconiosis patients compared with the general population. When adjusting for age, the annual mortality rate for the GoLBeT population was 22.45 (95% CI 12.56 to 37.02) per 1000 population. Age-adjusted SMRs further suggest higher mortality rates among all ages of adult podoconiosis patients (Table 1). The overall SMR for the study period was 5.99 (95% CI 3.61 to 9.36). The SMR for those ≤60 y of age was 12.42 (95% CI 6.79 to 20.84) and the SMR for those >60 y was 2.45 (95% CI 0.79 to 5.70).

**Table 1.** A summary of the age-adjusted annual mortality data and the associated SMRs for the GoLBeT and Dabat HDSS datasets

	Annual mortality rate per 1000 population (95% CI)		
Age group (years)	Dabat HDSS	GoLBeT	SMR (95% CI)
18–30	0.86 (0.56 to 1.25)	14.29 (0.36 to 79.59)	16.69 (0.42 to 93.00)
31–40	1.21 (0.68 to 2.00)	26.79 (5.52 to 78.28)	22.11 (4.56 to 64.62)
41–50	2.42 (1.46 to 3.78)	25.16 (6.86 to 64.42)	10.38 (2.83 to 26.57)
51–60	3.33 (2.00 to 5.19)	36.59 (13.43 to 79.63)	10.99 (4.03 to 23.93)
61–70	7.52 (4.95 to 10.94)	18.35 (2.22 to 66.28)	2.44 (0.30 to 8.81)
≥71	24.99 (19.45 to 31.63)	61.22 (12.62 to 178.92)	2.46 (0.51 to 7.16)

The age-adjusted SMRs provided very strong evidence for differences in mortality between populations in the 31–40, 41–50 and 51–60 y groups and no strong evidence for differences in the 18–30, 61–70 or ≥71 y age groups. This suggests that podoconiosis contributes to mortality, directly or indirectly. We found no evidence for trends in mortality by either disease stage or income, but numbers in the GoLBeT dataset were small. Podoconiosis patients may suffer sepsis secondary to adenolymphangitis or podoconiosis could interact with underlying comorbidities. Of the podoconiosis patients studied, the most common perimortem signs and symptoms included a feeling of weakness (31.6%), generalised swelling (26.3%), jaundice (15.8%) and difficulty breathing/shortness of breath (15.8%). Some of these signs are indicative of sepsis and/or organ damage, but without autopsy data this cannot be confirmed. The high prevalence of mental health disorders and poverty among patients might also be implicated in mortality.

Our study had a number of limitations. Both populations used family-reported dates of death, which could have introduced recall bias. The number of deaths in the GoLBeT dataset was small, making further mortality modelling adjustments inappropriate. Confounding may have been introduced due to differences in mortality between the datasets unrelated to podoconiosis.

## Conclusions

This study provides evidence that podoconiosis patients experience greater mortality than the general population in northern Ethiopia. If such an observation is confirmed in future studies, this will have important implications for our understanding of the global DALYs burden of podoconiosis, its impact on the health of individuals and the benefits on mortality of scaling up morbidity management programmes.

## Supplementary Material

traa150_Supplementary_File

## Data Availability

The Dabat HDSS is available via licensed access in the INDEPTH Data repository at https://www.indepth-ishare.org/index.php/catalog/163 (reference ID INDEPTH.ET051.CMD2015.V1). Data from the GoLBeT are available to researchers under the terms of the GoLBeT Data Sharing Protocol on the public webpage of the GoLBeT (https://podo.org/research/golbet-trial).
