# Supplementary material for: Excess mortality among people with podoconiosis: secondary analysis of two Ethiopian cohorts
Source: Trans R Soc Trop Med Hyg. 2020 Nov 24;114(12):1035–7. doi: 10.1093/trstmh/traa150 (PMC7738655; doi:10.1093/trstmh/traa150)
Supplement: traa150_Supplementary_File [file traa150_supplementary_file.docx]

**Supplementary Table 1**: Demographic data and the associated number of deaths for the GoLBeT and Dabat HDSS datasets, from the period 28^th^ February 2015-30^th^ July 2016

|  |  |  |  |  |  |  |  |  |  |  |
| --- | --- | --- | --- | --- | --- | --- | --- | --- | --- | --- |
|  | **GoLBeT** | | | | |  | **Dabat HDSS** | | | |
|  | Frequency (n) | | Percentage (%) | Number of deaths | |  | Frequency (n) | Percentage (%) | Number of deaths | |
|  |  | |  | n | % |  |  |  | n | % |
| **Sex** |  | |  |  |  |  |  |  |  |  |
| Male | 344 | | 51.9 | 8 | 42.1 |  | 20,927 | 47.5 | 76 | 43.4 |
| Female | 319 | | 48.1 | 11 | 57.9 |  | 23,168 | 52.5 | 99 | 56.6 |
| Total | 663 | | 100 | 19 | 100 |  | 44,095 | 100 | 175 | 100 |
| **Age** |  | |  |  |  |  |  |  |  |  |
| 18-30 years | 70 | | 10.6 | 1 | 5.3 |  | 21,379 | 48.5 | 26 | 14.9 |
| 31-40 years | 112 | | 16.9 | 3 | 15.8 |  | 8714 | 19.8 | 15 | 8.6 |
| 41-50 years | 159 | | 24.0 | 4 | 21.1 |  | 5515 | 12.5 | 19 | 10.9 |
| 51-60 years | 164 | | 24.7 | 6 | 31.6 |  | 4018 | 9.1 | 19 | 10.9 |
| 61-70 years | 109 | | 16.4 | 2 | 10.5 |  | 2526 | 5.7 | 27 | 15.4 |
| 71 and over | 49 | | 7.4 | 3 | 15.8 |  | 1943 | 4.4 | 69 | 39.4 |
| Total | 663 | | 100 | 19 | 100 |  | 44,095 | 100 | 175 | 100 |
